# Severe Climate‐Driven Range Contraction of *Taverniera abyssinica* A. Rich, an Endangered and Locally Popular Medicinal Plant in Ethiopia

**DOI:** 10.1002/ece3.73950

**Published:** 2026-07-01

**Authors:** Liyew Birhanu, Nigussie Amsalu, Dabash Tamir, Gerefa Sefu, Desalegn Chala

**Affiliations:** ^1^ Department of Biological Sciences, Faculty of Science, Royal Holloway University of London Egham UK; ^2^ Department of Biology Debre Markos University Debre Markos Ethiopia; ^3^ Jiangsu University Zhenjiang China; ^4^ Natural History Museum University of Oslo Oslo Norway

**Keywords:** climate change, conservation planning, Ethiopia, species distribution modeling, *Taverniera abyssinica*, traditional medicine

## Abstract

*Taverniera abyssinica* A. Rich. is one of the most important and well‐known medicinal plants used by local communities in Ethiopia. This critically endangered shrub is traditionally used to treat stomach ulcers, fever, soreness, and pain, and it is widely valued for its antipyretic and analgesic properties. In this study, we applied ensemble species distribution models using four algorithms (BRT, RF, GAM, GLM), 10‐fold cross‐validation, and three model replicates to predict the current and future distribution of *T. abyssinica* across Ethiopia. Suitable habitats were mapped at the district level to facilitate conservation planning and practical applications. Model performance was consistently high, with mean AUC values of 0.90–0.93 and TSS values exceeding 0.8, indicating strong predictive accuracy. Under current climatic conditions, the potential distribution of *T. abyssinica* is primarily concentrated in the central highlands, with additional suitable areas in the northern and eastern highlands. However, future climate projections indicate substantial range contraction, with approximately 85.9% (114,868 km^2^) of currently suitable habitat projected to become unsuitable, while only 2.7% (3573 km^2^) of new habitat is expected to become suitable. This represents a net habitat loss of 83.2% (111,295 km^2^). These findings indicate the vulnerability of *T. abyssinica* to climate change and underscore the urgent need for adaptive conservation strategies. Although model performance was robust, predictions are based on a relatively small number of occurrence records, meaning they capture only what is represented in the data. We therefore recommend that both current and projected future suitable areas be given conservation priority to safeguard this species.

## Graphical abstract

*Taverniera abyssinica*, a critically endangered Ethiopian medicinal plant, faces severe habitat loss due to climate change. Using ensemble species distribution models (BRT, RF, GAM, GLM), we predict an 83.2% net reduction in suitable habitat under future climate scenarios. Our findings highlight the urgent need for climate‐informed conservation strategies to protect this culturally and medically important species.

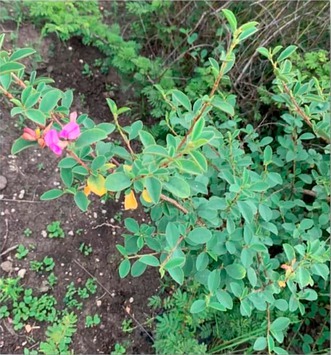

## 1 Introduction

Medicinal plants have a long history among humans and are the source of immediate medical care for 70%–95% of people in most developing countries (World Health Organization (WHO) 2002; Robinson and Zhang 2011). Beyond these developing regions, the use of medicinal plants is also steadily rising within wealthier, industrialized countries (Schippmann et al. 2002; Robinson and Zhang 2011). As of a 2016 assessment, an estimated 50,000 to 80,000 plant species globally are utilized for therapeutic purposes (Chen et al. 2016). This profound and widespread human reliance positions medicinal plants as flagship species, ideal for promoting biodiversity conservation and raising public awareness (Qian et al. 2021). Among the world's indigenous medical systems, African traditional medicine stands out as the oldest and arguably most diverse (Abdullahi 2011). Despite rapid advancements in conventional medicine, herbal remedies remain highly popular across the continent (Ozioma and Chinwe 2019). This enduring reliance highlights the critical role medicinal plants play in achieving the United Nations Sustainable Development Goals (SDGs), particularly SDG 3: ensuring healthy lives (Sharrock and Wyse Jackson 2017; Allievi et al. 2019).

Mirroring these global and regional trends, 80%–90% of the Ethiopian population relies on traditional medicine, largely based on medicinal plants for primary healthcare (Abebe 1986; World Health Organization (WHO) 2002; Kassaye et al. 2006). Driven by limited access to conventional healthcare, high pharmaceutical costs, and the widespread availability of plant‐based remedies (Yirga 2010; Lulekal et al. 2013), Ethiopia's deep‐rooted, often described as magico‐religious tradition uniquely integrates medical practices with spiritual and cultural beliefs (World Health Organization (WHO) 2002; Kassaye et al. 2006). This rich ethnobotanical heritage not only serves domestic needs but also represents a critical resource for global drug discovery (Hamilton 2004). However, Ethiopia's medicinal flora and its associated local knowledge face severe threats from anthropogenic pressures, including deforestation, overexploitation, overgrazing, habitat degradation, and agricultural expansion (Lulekal et al. 2013; Wasie 2020; Tafesse et al. 2023; Amsalu et al. 2025). Climate change further exacerbates these risks by restricting or shifting suitable habitats for many species. Consequently, many endemic plants are facing multiple threats; for example, *Lobelia rhynchopetalum* faces climate‐driven extinction, whereas *Echinops kebericho* is heavily depleted due to targeted overharvesting (Vivero et al. 2005; Chala et al. 2016; Tafesse et al. 2023).

Among Ethiopia's threatened medicinal flora, *T. abyssinica* A. Rich., locally known as “Dingetegna” (meaning “cure for sudden/acute illness”) and a member of the Fabaceae family, faces particularly considerable conservation challenges. Its widespread traditional use for ailments like fever, stomachaches, headaches, and “evil spirit” induced sudden illness fuels its overharvesting (Kloos et al. 2016; Gelan 2015). Given its critically endangered status (Vivero et al. 2005) and recognized medicinal and economic values, the conservation of *T. abyssinica* represents a top biodiversity priority for Ethiopia.

The primary therapeutic component is the roots, which are frequently chewed fresh or made as decoctions to treat pain, fever, and gastrointestinal spasms (Dagne et al. 1990; Noamesi et al. 1990). Other plant parts are utilized less frequently: stem bark is applied as a supportive remedy for general weakness and abdominal discomfort, while leaves are used in infusions for fever and headache relief; flowers are rarely reported in traditional use but may occasionally be included in multi‐component preparations (Mekuriaw 2010; Seifu et al. 2023). Root extracts have been used traditionally because pharmacological research has shown that they have strong antipyretic, analgesic, and antispasmodic properties (Dagne et al. 1990; Noamesi et al. 1990). Phytochemical evidence suggests that a variety of secondary metabolites, including isoflavonoids, flavonoids, phenolic compounds, alkaloids, tannins, saponins, and terpenoids, are mostly essential for these bioactivities. Together, these components support the plant's antibacterial, antioxidant, anti‐inflammatory, and smooth‐muscle‐relaxant properties, providing a scientific basis for its ethnomedical applications (Stadler et al. 1994; Mekuriaw 2010; Seifu et al. 2023). Overharvesting has been greatly aggravated by Ethiopia's heavy reliance on the medicinal value of its roots, raising concerns about conservation. Despite its significance, current conservation efforts for this species remain limited, hampered by poorly documented precise distribution and abundance. Cultivation offers a viable conservation strategy for endangered or over‐exploited medicinal plants (Sintayehu and Workineh 2020), and *T. abyssinica* has been specifically recommended for such ex‐situ conservation efforts (Asmelash et al. 2022) to safeguard this vital part of Ethiopia's threatened medicinal heritage.

Compounding these existing pressures, the pervasive phenomenon of climate change is projected to interact additively, synergistically, and potentially catastrophically with existing threats to medicinal plant species globally. Alterations in temperature and precipitation patterns, often influenced by shifting wind patterns, are known to significantly affect plant growth and their biological activity (Ayob et al. 2017). Consequently, many plant species are predicted to respond to ongoing climate change by shifting their geographical ranges or facing extinction soon (Mahmoud et al. 2017). Furthermore, various environmental stresses, including temperature extremes, elevated CO_2_, and ozone significantly impact the production of plant secondary metabolites. These compounds are crucial for plant adaptation, often playing a vital role in their defense mechanisms and, importantly, possessing the therapeutic properties for which medicinal plants are valued. This critical knowledge gap necessitates further study to fully understand and harness their therapeutic value in a rapidly changing global climate (Jangpangi et al. 2025). This pressing need for more research directly impacts conservation strategies, especially for vulnerable species like Ethiopia's *T. abyssinica* .

To address this critical knowledge deficit, Species Distribution Modeling (SDM) is a widely utilized ecological tool for predicting and estimating the potential effects of climatic changes and informing conservation planning (Sofi and Saba 2022; Tafesse et al. 2023). While several studies have employed SDMs to model the geographical distribution of threatened and endemic medicinal plants, direct research focusing on the impacts of climate change on *Taverniera* species within Ethiopia remains absent. Previous studies on *T. abyssinica* have focused on its antimicrobial activity (Buli et al. 2015), the identification of isoflavonoid compounds (Duddeck et al. 1987), in vitro regeneration techniques (Gelan 2015), and its agronomic requirements (Wube and Atnafu 2022). However, a significant gap exists in understanding the impacts of climate change on its distribution.

Given that *T. abyssinica* is geographically restricted, exhibits poor natural regeneration (Asmelash et al. 2022), is classified as critically endangered in Ethiopia (Vivero et al. 2005), and is considered vulnerable to climate change (Wakie et al. 2014), this study aims to model its current and future potential distribution under different climate change scenarios. Specifically, we test the hypotheses that (i) the climatically suitable area for *T. abyssinica* is larger than its currently documented distribution, indicating the presence of potentially undiscovered suitable habitats, and (ii) future climate change will significantly alter the extent and spatial distribution of suitable habitats, with implications for the species' long‐term conservation.

## 2 Methodology

### 2.1 Description of Study Area

Ethiopia, situated in the Horn of Africa between 3°–15° N and 33°–48° E, shares borders with Kenya (south), Somalia (southeast), Djibouti (east), Eritrea (north), Sudan (northwest), and South Sudan (west). Covering approximately 1.14 million km^2^, the country features a landscape dominated by high, rugged plateaus and surrounding arid and semi‐arid lowlands (Chala et al. 2017).

### 2.2 Description of *Taverniera abyssinica*

**FIGURE 1:**
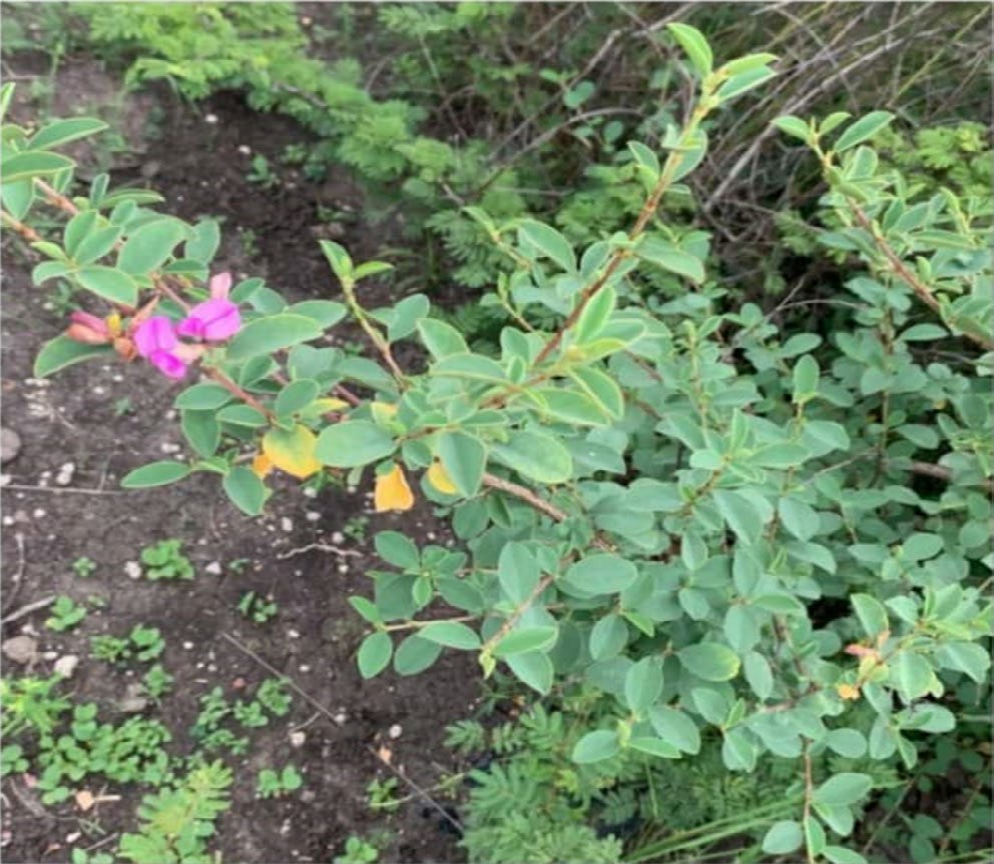
Habit of *Taverniera abyssinica* A. Rich., observed at Shewa floristic region, Ethiopia (Asmelash et al. 2022).

*Taverniera abyssinica*, a highly threatened medicinal plant (Vivero et al. 2005), is typically found in specific bushland limestone regions of Ethiopia. It thrives at altitudes ranging from 1700 to 2300 m above sea level (Abera 2014). This species presents as a pinnately three‐foliolate shrub or shrublet, reaching up to 2 m in height. Its leaflets are smooth (glabrous) on the upper surface but covered with appressed hairs (appressed pubescent) underneath. The plant produces racemes with 2 to 8 flowers, and the combined length of its rhachis and peduncle measures approximately 3–25 mm. The calyx is outwardly appressed pubescent, with lobes that are equal to or longer than the tube. Its striking corolla, measuring 12–17 mm long, ranges in color from dark pink to purplish pink (Figure 1). *Taverniera abyssinica* was identified using diagnostic morphological and floral characters based on the taxonomic keys provided in the Flora of Ethiopia and Eritrea (Thulin 1989).

**FIGURE 2:**
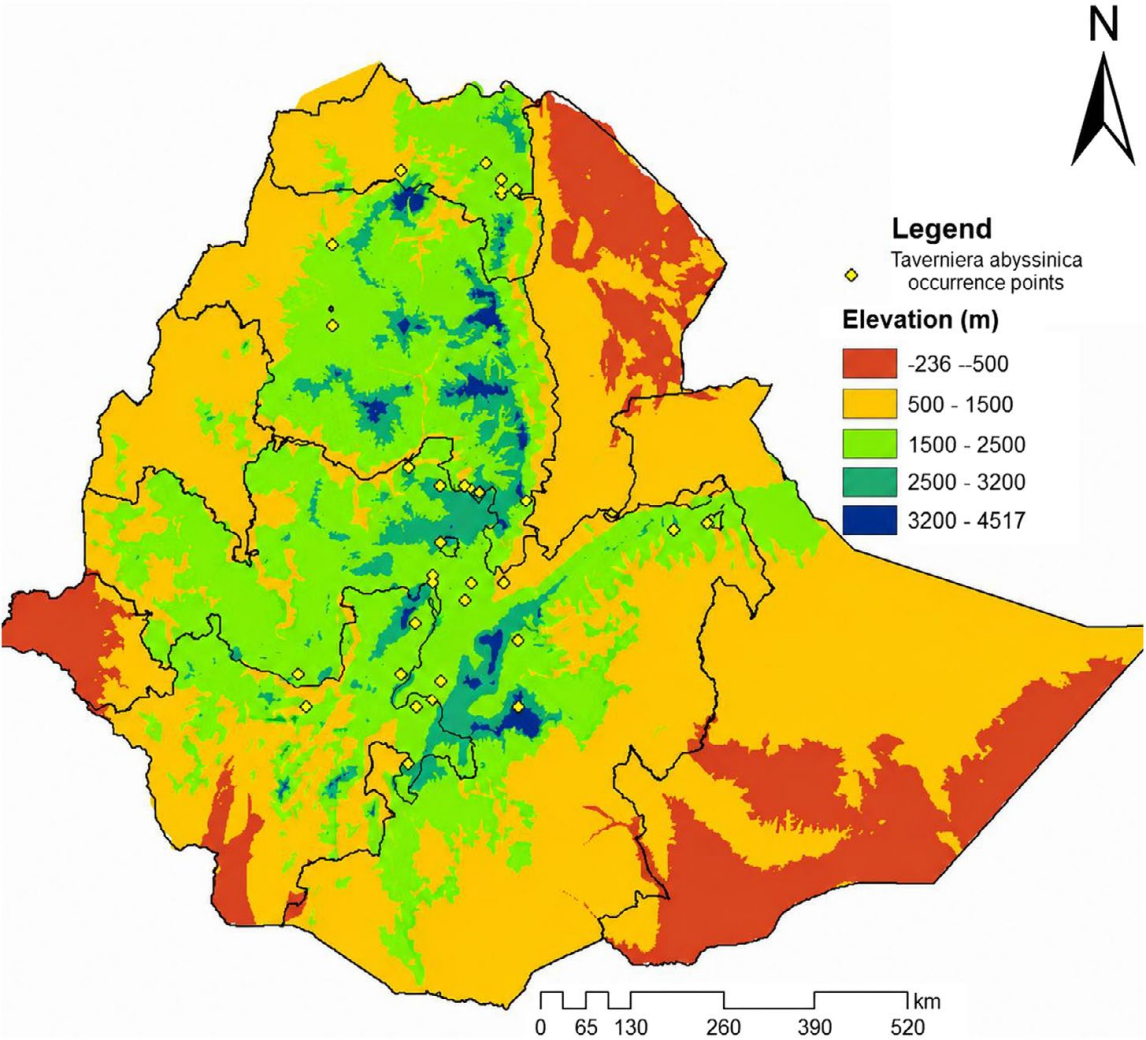
Occurrence points of *Taverniera abyssinica* used in the study (the study area map was prepared using shapefile data from the Ethiopian Mapping Agency shapefiles. Occurrence points of *T. abyssinica* were obtained from field surveys, published literature, and GBIF).

Ethnobotanical records highlight the significant historical use of *T. abyssinica* in Ethiopian traditional medicine for various ailments. The genus *Taverniera* is particularly noted in rural parts of Ethiopia for treating diverse conditions, including stomach ulcers, providing immediate relief from sudden illnesses and fever, and alleviating soreness and pain, functioning locally as an antipyretic and analgesic (Buli et al. 2015). Unfortunately, *T. abyssinica* is now only found as remnant, scattered populations in the Shewa, Tigray, and Wollo regions of Ethiopia (Figure 2). Prior to data collection, a letter of consent and permission was obtained from Debre Markos University.

### 2.3 Climate Data Acquisition

To model the potential distribution of *T. abyssinica* , we utilized both current and future climate data. The current climate data comprised 19 bioclimatic variables at a spatial resolution of 1 km, obtained from the WorldClim 2.1 database (Fick and Hijmans 2017). Future climate projections were derived from the CMIP6 framework using the ACCESS‐CM2 model, based on two scenarios: SSP1‐2.6 (future_126) and SSP5‐8.5 (future_585), covering the period 2061–2080. These datasets had the same resolution as the current climate data. All climate data were clipped to the geographic boundary of Ethiopia.

### 2.4 Occurrence Data and Background Points

Species occurrence records for *T. abyssinica* were compiled from field surveys, previously published literature, and the Global Biodiversity Information Facility (GBIF) database (GBIF 2025). To mitigate sampling bias and spatial autocorrelation, the records were spatially rarefied using a minimum distance threshold of 1 km, implemented via the spThin package in R (Aiello‐Lammens et al. 2015). After all data cleaning and spatial thinning procedures, a total of 31 occurrence records were retained and used for modeling. To enhance model robustness, 10,000 background points were randomly generated across the study area. Climatic values were then extracted for both occurrence and background points for subsequent analysis.

### 2.5 Variable Selection

To address multicollinearity among predictor variables, current climate variables were extracted at both occurrence and randomly generated background points. A Pearson pairwise correlation analysis was conducted, and for pairs of variables with a correlation coefficient ≥ |0.8|, only a variable with the lowest Variance Inflation Factor (VIF) was retained using the “vifcor” function from the “usdm” R package (Naimi et al. 2014). As a result, 11 out of the original 19 bioclimatic variables were selected and used for model runs: bio2 (Mean Diurnal Range), bio3 (Isothermality), bio4 (Temperature Seasonality), bio7 (Temperature Annual Range), bio9 (Mean Temperature of Driest Quarter), bio12 (Annual Precipitation), bio13 (Precipitation of Wettest Month), bio14 (Precipitation of Driest Month), bio15 (Precipitation Seasonality), bio18 (Precipitation of Warmest Quarter), and bio19 (Precipitation of Coldest Quarter).

### 2.6 Modeling

Species distribution models (SDMs) were developed using the sdm package in R, employing four algorithms: Boosted Regression Tree (BRT), Random Forest (RF), Generalized Additive Models (GAM), and Generalized Linear Models (GLM). A 10‐fold cross‐validation approach was implemented, with three replications, resulting in a total of 120 models (4 algorithms × 10 folds × 3 replications). Parallel computing with 24 cores was utilized to enhance computational efficiency.

Model performance was evaluated using the True Skill Statistic (TSS) and the Area Under the Receiver Operating Characteristic Curve (AUC) (Liu et al. 2013). Models with AUC > 0.7 and TSS > 0.4 were considered for further analysis, yielding 116 models that met the criteria. These models were then projected onto future climate scenarios under two Shared Socioeconomic Pathways (SSPs) using ensemble species distribution modeling approaches (Thuiller et al. 2019; Luo et al. 2023).

Predicted probability maps were converted into binary suitability maps (suitable/unsuitable) using two threshold criteria: the maximum sum of sensitivity and specificity and the 10% presence threshold. This process generated 232 binary maps (116 models × 2 thresholds) across current and future climate scenarios.

To produce final suitability maps, the binary maps were aggregated and classified into three suitability classes: Unsuitable (0–0.3)—pixels where 69 or fewer binary maps predicted presence; uncertain (> 0.3–0.6)—pixels where 70–139 binary maps predicted presence; suitable (> 0.6)—pixels where 140 or more binary maps predicted presence (Chala et al. 2016). An overlay of the suitability classes was compared to detect changes in suitable habitats between the current and future climates. For future climates, the average of the two emission scenarios was used, resulting in the generation of 464 binary maps and classifying following a similar method.

## 3 Results

### 3.1 Evaluation of Model Prediction Accuracy

Our models demonstrated a very high performance. The average AUC values across model runs ranged from 0.90 to 0.93 for all algorithms, indicating excellent model discrimination ability. TSS scores were consistently above 0.8, reflecting high sensitivity and specificity in model predictions. Deviance values were low across all models, while correlation values remained within an acceptable range (Table 1).

**TABLE 1:** Performance evaluation of SDMs using different statistical parameters the ensemble model.

Species	Methods	AUC	COR	TSS	Deviance
*Taverniera abyssinica*	BRT	0.92	0.12	0.86	0.03
	RF	0.9	0.13	0.82	0.04
	GAM	0.93	0.14	0.86	0.04
	GLM	0.9	0.09	0.82	0.03
	Mean	0.91	0.12	0.84	0.035

### 3.2 Current and Future Distributions of *Taverniera abyssinica*

**FIGURE 3:**
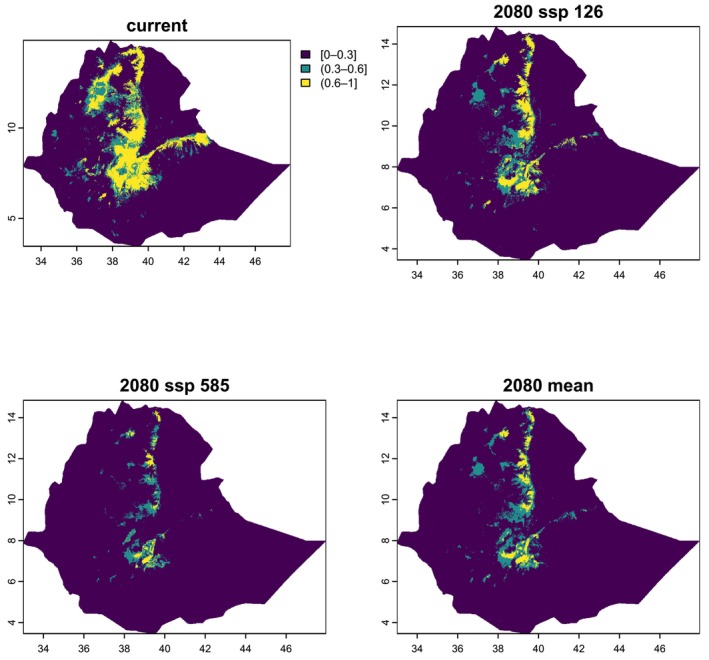
Predicted habitat suitability of *Taverniera abyssinica* in Ethiopia under current and future climate condition, based on 232 binary maps. Areas classified as unsuitable (suitability score 0–0.3) are pixels where 69 or fewer binary maps predicted species presence; uncertain areas (> 0.3–0.6) are pixels where 70 to 139 binary maps predicted presence; and suitable areas (> 0.6) are pixels where 140 or more binary maps predicted presence. The 2080 SSP1‐2.6 map represents habitat suitability by 2080 under the lowest emission scenario, while the 2080 SSP5‐8.5 map reflects suitability under the worst‐case emission scenario. The final map presents the mean suitability derived from the two future scenarios (from 464 binary habitat suitability maps generated using the same classification criteria).

Under the current climate scenario, the suitability habitat of *T. abyssinica* was mainly concentrated in the central highlands region of Ethiopia. Additional suitable areas are predicted in the northern regions, including Tigray and the Amhara regions. The species is predicted to occur in the eastern highlands of Ethiopia. Smaller pockets of suitable habitats are predicted to be found in South‐Central Ethiopia (Figure 3).

**FIGURE 4:**
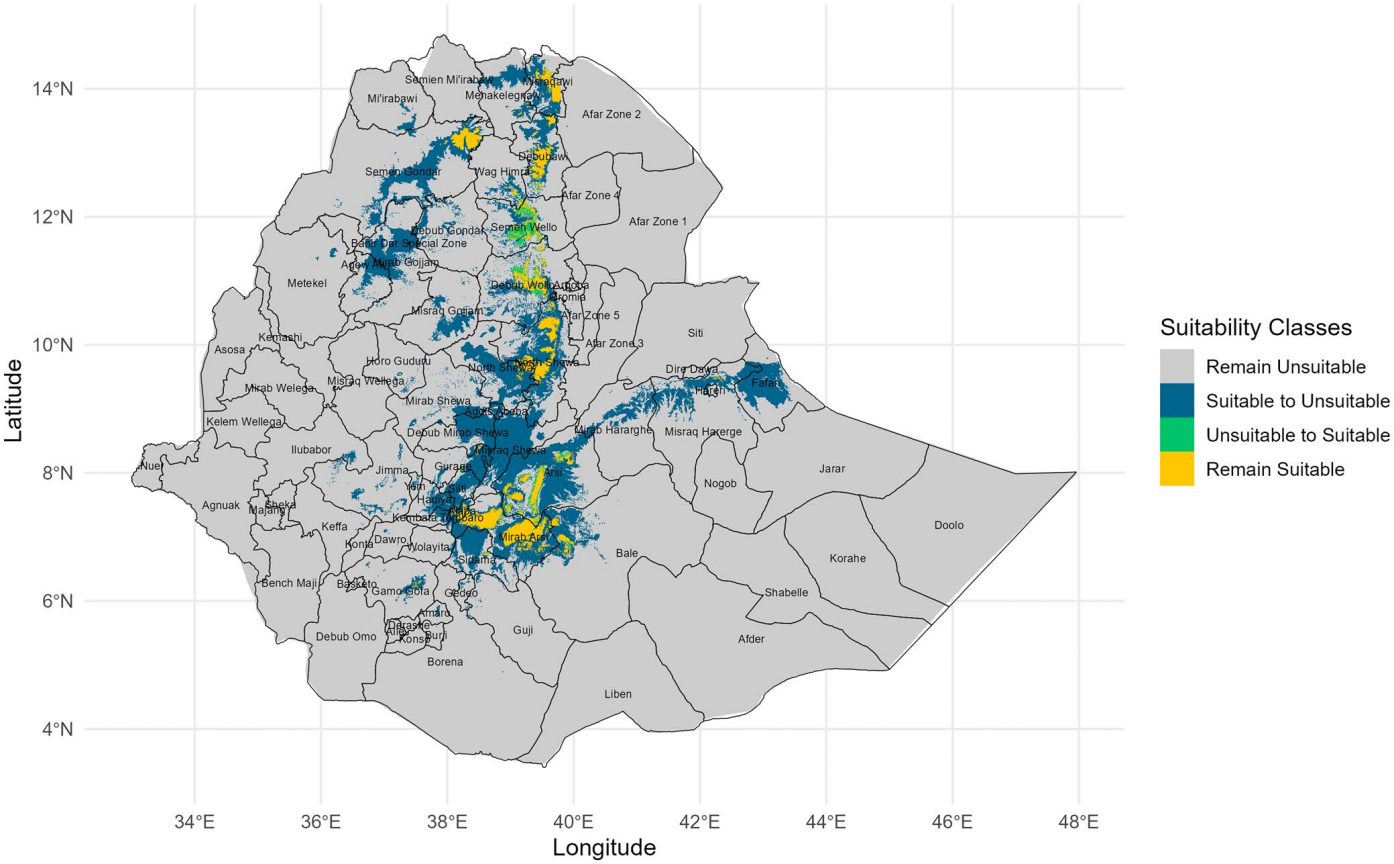
Projected changes of habitat suitability of *Taverniera abyssinica* distribution between current and future climate model, overlaid with the administrative zones of Ethiopia. “Debub” means south, “Semen” means north, “Misrak” means east, and “Mi'irab” means west in Amharic. Suitable to unsuitable indicates areas currently suitable that are projected to become unsuitable under future climate conditions (habitat loss). “Unsuitable to suitable” represents areas currently unsuitable that are projected to become suitable in the future (potential habitat gain and species shift). “Remain suitable” indicates areas that are projected to remain suitable under both current and future climates.

The suitable habitats of *T. abyssinica* are projected to decrease significantly under future climate scenarios (2080 SSP1‐2.6 and SSP5‐8.5), especially under the more extreme SSP5‐8.5 scenario (Figure 3). Approximately 85.9% (114,868 km^2^) of currently suitable areas are projected to become unsuitable, while only 2.7% (3573 km^2^) of new areas are expected to become suitable. About 83.2% (18,822 km^2^) of the current suitable habitat is predicted to remain suitable despite climate change (Table 2, Figure 4).

**TABLE 2:** Changes in habitat suitability for *Taverniera abyssinica* under climate change.

Change	Area (km^2^)
Suitable to unsuitable	114,868
Unsuitable to suitable	3573
Remain suitable	18,822

This leads to a net habitat loss of approximately 83.2% (111,295 km^2^), highlighting a significant contraction in the species' potential range and emphasizing the urgent need for targeted conservation efforts.

## 4 Discussion

This study provides the first assessment of the current and future potential distribution of *T. abyssinica* under climate change scenarios in Ethiopia. Our results support the hypotheses that (i) the climatically suitable area for the species extends beyond its currently documented distribution and (ii) future climate change is likely to substantially reduce and alter the distribution of suitable habitats. Under current climatic conditions, suitable habitats are concentrated in the central Ethiopian highlands, with additional fragmented areas occurring in the northern and eastern highlands. However, future climate projections indicate substantial habitat contraction, with more than 80% of currently suitable habitats projected to become unsuitable by the end of the century. These findings highlight the high vulnerability of *T. abyssinica* to climate change and emphasize the need for proactive conservation planning. The ensemble modeling framework used in this study provided robust and reliable predictions. The ensemble model reduces uncertainty related to model fitting by combining the predictions of several individual models (Thuiller 2014). This approach significantly improves the reliability of predictions compared to using individual models alone (Araújo and New 2007; Barbet‐Massin et al. 2012). The models used in this study achieved an excellent degree of accuracy, with mean AUC and TSS values of 0.91 and 0.84, respectively. Furthermore, despite generating 118 individual models under current climatic conditions, the variation among model predictions was minimal, demonstrating the robustness and stability of the modeling framework (Table 1).

The predicted distribution under the current climate conditions closely matches the documented occurrence of *T. abyssinica* in the central highlands of Ethiopia, in the western highlands of Amhara and Tigray as well as isolated pockets in south and eastern Ethiopian mountains (Vivero et al. 2005; Asmelash et al. 2022), providing further support for model reliability. Integrating field occurrence records, herbarium data, and predictive modeling creates a robust, multi‐dimensional view of the species' natural range boundaries (Scheldeman and van Zonneveld 2010). Importantly, our model also identified climatically suitable areas beyond the currently documented range, suggesting the possibility of undocumented populations or suitable habitats that remain unoccupied due to dispersal limitations, historical factors, or anthropogenic pressures (Figures 3 and 4). We recommend assisted establishment of the species in currently unoccupied, yet suitable habitats identified by our models as a potential strategy to enhance its long‐term persistence and conservation.

The magnitude of habitat loss projected in this study suggests that *T. abyssinica* is highly vulnerable to future climate change because more than 80% of current suitable habitats are expected to become unsuitable by mid‐ to late‐century. The projected contraction of suitable habitats under future climate scenarios is consistent with broader understanding that climate change is reshaping species' geographic distributions worldwide (Chala et al. 2017; Sales et al. 2020; Semu et al. 2021; Enkossa et al. 2022; Daba et al. 2023). Changes in land use can also reduce the availability of suitable regions that could host future populations of *T. abyssinica* (Asmelash et al. 2022).

The species prefers dry Afromontane and escarpment habitats, growing in stony, shallow soils, and degraded bushlands, primarily in the central and northern Ethiopian highlands (Asmelash et al. 2022). Its ecological niche is narrow and fragmented, which limits its ability to adapt or migrate under rapidly changing climatic conditions. This ecological specialization likely contributes to the limited persistence of suitable habitats under future climates, with only approximately 14% of the current suitable areas projected to remain as climatic refugia, mainly in the central highland areas.

The distribution modeling of highland species such as *Prunus africana* a widely harvested medicinal tree predicts strong contractions of suitable habitat in East Africa, driven by both climate change and unsustainable harvesting (Mbatudde et al. 2012; Giliba and Yengoh 2020). In South Africa, *Siphonochilus aethiopicus* an overexploited medicinal plant has experienced severe population decline due to combined climate pressures and human demand, with models indicating further contraction unless ex‐situ cultivation is expanded (Williams et al. 2013). Globally, studies on medicinal species show similar vulnerabilities: for instance, SDMs of *Taxus wallichiana* in the Himalayas (Kharkwal and Mehrotra 2020) and *Panax ginseng* in East Asia (Shin et al. 2018) also predict large range losses under warming scenarios, highlighting a common trend of climate sensitivity in medicinal species with restricted ecological niches.

Beyond its ecological vulnerability, *T. abyssinica* holds immense cultural and medicinal value, making its conservation an urgent priority. In traditional Ethiopian medicine, the species is highly valued for its efficacy as an antipyretic (fever reducer) and analgesic, commonly used to treat stomach ulcers, fevers, and acute pain (Dagne et al. 1990; Seifu et al. 2023). Because the majority of Ethiopia's population relies on traditional remedies (Abebe 1986; World Health Organization (WHO) 2002), the depletion of this species threatens not only biodiversity but also cultural heritage and local healthcare resilience. This vulnerability mirrors global patterns, where climate‐driven losses in medicinal flora jeopardize rural healthcare systems that depend directly on local ecosystems (Hamilton 2004). Conservation planning should therefore prioritize refugia identified in our model, protecting them through community‐managed reserves and integration into Ethiopia's ongoing land restoration programs. Ex‐situ approaches including nursery propagation, seed banking, and tissue culture are already feasible for *T. abyssinica* (Asmelash et al. 2022) and can complement in situ measures.

### 4.1 Limitations and Future Research Directions

Our models achieved high predictive accuracy (AUC = 0.91; TSS = 0.84 on average), and variation among the 116 individual model runs was minimal (Table 1), indicating a high degree of robustness despite the limited number of occurrence records available for the species. Nevertheless, some sources of uncertainty remain.

First, the study was geographically limited to Ethiopia, although *T. abyssinica* has recently been reported from other parts of the Horn of Africa and the southwestern Arabian Peninsula. Consequently, the model was calibrated using only a portion of the species' known distribution, and environmental conditions occurring outside Ethiopia may not be fully represented. Future studies incorporating data from the entire distribution range would allow a more comprehensive assessment of the species' ecological niche and improve the transferability of model predictions.

Second, the availability of documented occurrence records for *T. abyssinica* remains limited (GBIF 2025). Although model performance was high and uncertainty among model runs was low, sparse occurrence data can reduce the representation of rare environmental conditions and increase sensitivity to sampling biases (Ahmed et al. 2023). Additional records from field surveys, herbaria, citizen science initiatives, and biodiversity databases would improve confidence in estimates of habitat suitability and environmental responses. Expanded occurrence data would also facilitate a more detailed assessment of the relative importance of climatic and environmental drivers shaping the species' distribution.

Third, the modeling framework relied exclusively on climatic predictors and did not explicitly incorporate other potentially important determinants of species distribution, including land‐use change, agricultural expansion, deforestation, habitat fragmentation, grazing pressure, and soil characteristics. These factors may strongly influence the realized distribution and long‐term persistence of the species. Therefore, the predicted distributions should be interpreted as areas of potential climatic suitability rather than the realized distribution of *T. abyssinica* , which may be further constrained by anthropogenic and environmental factors.

Fourth, important ecological processes such as flowering phenology, pollination biology, seed dispersal mechanisms, and other biotic interactions were not included in the modeling framework. These factors can influence species establishment, persistence, dispersal, and responses to climate change (Anthelme and Dangles 2012; Inouye 2020; Richman et al. 2020). Furthermore, phenological patterns may vary across Ethiopia because rainfall regimes differ among regions and mountain systems, with some areas experiencing unimodal rainfall and others bimodal rainfall patterns. Such spatial variability makes it difficult to incorporate phenological information consistently at a national scale. Future studies should investigate the role of these ecological processes in shaping the distribution and climate‐change responses of *T. abyssinica* .

Despite these limitations, the consistency of model performance across cross‐validation runs and the low variation among ensemble predictions suggest that the broad patterns identified in this study are robust. We therefore recommend prioritizing the conservation of areas identified as suitable under both current and future climate conditions, particularly those projected to function as climatic refugia.

## 5 Conclusions

Species distribution modeling is a valuable tool for assessing the potential impact of climate change on species that require monitoring and management. Our results indicate that suitable habitat for *T. abyssinica* is primarily concentrated in the central Ethiopian highlands, with additional suitable areas in the northern and eastern highlands. Our findings show that *T. abyssinica* , a critically endangered medicinal plant in Ethiopia, is highly vulnerable to climate change. Our results indicate that approximately 83.2% (111,295 km^2^) of its currently suitable habitat could be lost under future climate scenarios, leaving only a small proportion of stable or newly emerging areas. To reduce the risk of further population decline, conservation efforts should combine both in situ and ex‐situ approaches. In situ conservation should prioritize the potential distribution areas identified under both current and future climates, establishing fenced micro‐reserves to prevent wild foraging and agricultural encroachment. Ex‐situ measures, including seed‐banking, propagation, and cultivation programs, could complement these efforts by reducing pressure on wild populations and safeguarding the species' genetic resources. Given the species' medicinal importance and cultural significance, its conservation is important not only for biodiversity preservation but also for maintaining traditional healthcare resources and associated local knowledge. Generally, areas identified as suitable under both current and future climate scenarios should be prioritized for conservation, restoration, and potential reintroduction efforts or assisted establishment programs, including integration into Ethiopia's Green Legacy Initiative. Similarly, climatically suitable but currently unoccupied areas may represent potential targets for future conservation translocations aimed at enhancing the long‐term persistence of the species.

## Author Contributions

**Liyew Birhanu:** conceptualization (equal), data curation (equal), formal analysis (equal), investigation (equal), methodology (equal), supervision (lead), validation (equal), visualization (equal), writing – original draft (equal), writing – review and editing (equal). **Nigussie Amsalu:** conceptualization (equal), methodology (equal), writing – original draft (equal), writing – review and editing (equal). **Dabash Tamir:** conceptualization (equal), data curation (lead), investigation (equal), visualization (equal), writing – original draft (equal), writing – review and editing (equal). **Gerefa Sefu:** methodology (equal), writing – review and editing (equal). **Desalegn Chala:** conceptualization (equal), data curation (equal), formal analysis (lead), investigation (equal), methodology (equal), resources (equal), software (lead), validation (equal), visualization (equal), writing – original draft (equal), writing – review and editing (equal).

## Funding

The authors have nothing to report.

## Conflicts of Interest

The authors declare no conflicts of interest.

## Supporting information


**Table S1:** Occurrence points of *Taverniera abyssinica* used in the study.

## Data Availability

The datasets used in this study are freely available from the following sources: Presence records of the 31 occurrence points of the species are given in Table S1. Bioclimatic data: WorldClim (https://www.worldclim.org).
