# Supplementary material for: Severe Climate‐Driven Range Contraction of Taverniera abyssinica A. Rich, an Endangered and Locally Popular Medicinal Plant in Ethiopia
Source: Ecol Evol. 2026 Jul 1;16(7):e73950. doi: 10.1002/ece3.73950 (PMC13321467; doi:10.1002/ece3.73950)
Supplement: Supplementary file 1 — Table S1: Occurrence points of Taverniera abyssinica used in the study. [file ECE3-16-e73950-s001.pdf]

Table s1 occurrence points of *Taverniera abyssinica* used in the study

	Species	Longitude	Latitude
1	Taverniera abyssinica	39	8.4
2	Taverniera abyssinica	38.6	8.6
3	Taverniera abyssinica	38.6	8.7
4	Taverniera abyssinica	39.5	13.5
5	Taverniera abyssinica	39.3	13.7
6	Taverniera abyssinica	39.7	13.4
7	Taverniera abyssinica	39.1	8.6
8	Taverniera abyssinica	39.5	13.3
9	Taverniera abyssinica	38.2	13.6
10	Taverniera abyssinica	39.2	9.7
11	Taverniera abyssinica	39.5	8.6
12	Taverniera abyssinica	39	9.8
13	Taverniera abyssinica	38.4	8.1
14	Taverniera abyssinica	39.8	9.6
15	Taverniera abyssinica	41.4	10.3
16	Taverniera abyssinica	38.3	10
17	Taverniera abyssinica	41.7	9.2
18	Taverniera abyssinica	38.7	7.4
19	Taverniera abyssinica	36.9	7.5
20	Taverniera abyssinica	37	7.1
21	Taverniera abyssinica	39.7	7.1
22	Taverniera abyssinica	39.7	7.9
23	Taverniera abyssinica	37.3	11.7
24	Taverniera abyssinica	38.2	7.5
25	Taverniera abyssinica	38.4	7.1
26	Taverniera abyssinica	38.6	7.2
27	Taverniera abyssinica	38.3	6.4
28	Taverniera abyssinica	37.3	12.7
29	Taverniera abyssinica	38.7	9.8
30	Taverniera abyssinica	38.7	9.1
31	Taverniera abyssinica	42.1	9.3
